# Financial Hardship in Dementia: Community Focus Group Findings on Measure Development

**DOI:** 10.1093/geroni/igaf122.3935

**Published:** 2025-12-31

**Authors:** Jacqueline Telonidis, Nicole Fleming, Kara Dassel, Jace Flatt, Hanna Hedges, Jia-Wen Guo, Eli Iacob, Sara Bybee

**Affiliations:** University of Utah, Salt Lake City, Utah, United States; University of Utah, Salt Lake City, Utah, United States; University of Utah, Salt Lake City, Utah, United States; University of Nevada Las Vegas, Nevada, United States; University of Utah, Salt Lake City, Utah, United States; University of Utah, Salt Lake City, Utah, United States; University of Utah, Salt Lake City, Utah, United States; University of Utah, Salt Lake City, Utah, United States

## Abstract

By 2060, nearly 14 million Americans will live with dementia. As one of the most expensive diseases, dementia costs primarily burden persons living with dementia (PLWD) and their care partners. Given the association between financial hardship and worsening health, this study aimed to develop and validate an inclusive dementia-focused measure of financial hardship for LGBTQ+ and non-LGBTQ+ PLWD and their care partners. We conducted two 90-minute focus groups with aging and/or LGBTQ+ health professionals (n = 7)—including social workers, researchers, and nurses—and non-LGBTQ+ PLWD and their care partners (n = 8). The LGBTQ+ PLWD focus group remains to be conducted. Using the Financial Hardship Typology framework, we examined material costs, emotional responses, and coping behaviors. Participants reviewed 27 potential items adapted from the COmprehensive Score for financial Toxicity (COST) used in cancer care, discussing each item’s relevance to financial hardship and quality of life using a 4-point Likert scale. Focus groups were audio recorded, transcribed verbatim, and analyzed using qualitative content analysis. Ratings were summarized with descriptive statistics. The most relevant items included: using savings for medical care, worrying about affording care, worrying if income covers care costs, and distress about future financial security. Both groups recommended separate measures for care partners and PLWD based on disease severity. Dyads emphasized psychological distress from dementia-related financial hardships. These findings will inform development of a more feasible, accessible, and useable financial hardship measure for PLWD and their care partners.

